# Gasping for air

**DOI:** 10.7554/eLife.27004

**Published:** 2017-05-02

**Authors:** Steven G Ball, Ugo Cenci

**Affiliations:** Institute for Functional and Structural Glycobiology (UGSF), UMR8576 University of Lille/CNRS, Villeneuve d’Ascq, Francesteven.ball@univ-lille1.fr; Institute for Functional and Structural Glycobiology (UGSF), UMR8576 University of Lille/CNRS, Villeneuve d’Ascq, France

**Keywords:** symbiosis, RNA-seq, transcriptomics, endosymbiosis, Oophila amblystomatis, Ambystoma maculatum, Other

## Abstract

Transcriptomics is shedding new light on the relationship between photosynthetic algae and salamander eggs.

**Related research article** Burns JA, Zhang H, Hill E, Kim E, Kerney R. 2017. Transcriptome analysis illuminates the nature of the intracellular interaction in a vertebrate-algal symbiosis. *eLife* **6**:e22054. doi: 10.7554/eLife.22054

Primary endosymbiosis involves the penetration of single cell bacteria into the cytoplasm of eukaryotic host cells, whereas secondary endosymbiosis involves single cell eukaryotes entering eukaryotic host cells. Both of these forms of endosymbiosis are usually followed by many different types of symbiotic interactions. Moreover, certain organelles found in eukaryotic cells, notably mitochondria and plastids, are the result of endosymbiosis.

Endosymbiosis is rather rare in vertebrates, mainly because their immune system is likely to fend off any invaders. So what challenges do potential symbionts face upon entering a potentially hostile intracellular environment? Now, in eLife, John Burns of the American Museum of Natural History, Ryan Kerney of Gettysburg College and colleagues provide a surprising answer to these questions (Burns et al., 2017).

A well-known example of a symbiotic relationship is that of the green Volvocean alga *Oophila amblystomatis*, and the spotted salamander *Ambystoma maculatum*. These algae are photosynthetic, so they use light energy to produce sugar and oxygen. *Oophila* algae also need oxygen to survive, but they can withstand anoxia (that is, a total lack of oxygen) for short periods of time.

It has been shown that *Oophila* algae grow better in water populated with salamander embryos, and the salamander embryos are healthier when more algae are present. Initially, it was thought that the alga and the salamander form an ectosymbiotic relationship, where the alga grows around the eggs of the salamander and supplies the embryo with oxygen and sugar in exchange for waste products (Graham et al., 2013; Small et al., 2014). However, it was recently discovered that these ectosymbionts can reach and penetrate into embryo cells to also form an endosymbiotic relationship, with the alga living inside the embryo cells of the salamander (Kerney et al., 2011).

To better understand the molecular mechanisms underlying this relationship, Burns et al. used a technique called differential expression RNA-seq analysis to compare the transcriptomes (that is, all the gene transcripts) of three different types of algae: ectosymbiotic algae that lived around the egg; endosymbiotic algae that lived inside the embryos; and algae that were grown in the laboratory (Burns et al., 2017). In addition, they compared the transcriptomes of the salamander embryo cells that either contained or lacked the algae.

The technical originality and finesse of this approach lie in the comparison between ecto- and endosymbiotic transcriptomes of the algae: studying the different types of symbiotic relationships that occur within the same individual helps to provide a clearer picture of the molecular mechanisms that shape these processes.

Burns et al. discovered that the endosymbiotic algae experienced a shift from an oxidative metabolism to a fermentative metabolism. The algae also experienced a clear increase in transcripts from genes of algal fermentative metabolism, including an increase of hydrogenase, which is the hallmark of a switch to anoxia (Grossman et al., 2011). This was accompanied by a reduction in transcripts of core components of photosystem II (which produces oxygen) and mitochondrial complex I. This response mirrors the response of the related green alga *Chlamydomonas reinhardti* to an absence of sulfur, which involves a reduction in oxygen production by photosystem II, as well as reductions in sulfate transport and sulfur metabolism (Nguyen et al., 2008).

The limitations imposed on photosynthesis by shortages of oxygen and/or sulfur, combined with a lack of light inside the embryonic cells, means that very little carbon fixation will occur. This is consistent with previous work which showed that immotile endosymbionts display lower levels of starch than free-swimming ectosymbionts, despite the fact that swimming has been demonstrated to have a strong negative impact on starch accumulation in *C. reinhardti* (Hamilton et al., 1992).

In addition to those symptoms related to a lack of oxygen, the algae inside the embryo showed signs of cellular stress and had higher levels of proteins that are usually expressed in response to stressors such as heat shock or autophagy. Hence, the endosymbionts are likely gasping for air and actively breaking down their polysaccharide stores by fermentation, which is a well-known response to hypoxia in Volvocean algae (Klein and Betz, 1978). Given these circumstances, it seems rather unlikely that the endosymbiotic algae are in a position to supply significant amounts of either oxygen or photosynthate to the embryo cells. It remains thus unclear how these cells may actually benefit from the presence of the algae.

The dramatic response of the endosymbiotic algae to a lack of oxygen somewhat resembles the responses that occur in certain intracellular parasites (Polonais and Soldati-Favre, 2010) or bacteria. These microorganisms show similar stress responses and need fermentation to create energy. In addition, some bacteria use a specific enzyme with a high affinity to oxygen to initiate a cellular ‘microaerophilic’ response when oxygen is scarce or unavailable (Juul et al., 2007; Omsland et al., 2013).

The embryo cells, on the other hand, appeared to be rather unfazed by the algae living inside them. Modifications in their gene transcripts suggested a lowered innate immune response, and while the embryo cells with endosymbiotic algae experienced changes in their metabolic signaling pathways, they did not exhibit any signs of stress.

The work by Burns et al. highlights the problems a lack of oxygen in the intracellular environment can pose for photosynthetic algae, once they have managed to breach the immune defenses of their host. The unexpected shortage of sulfur inside the host cell exacerbates these problems, leading to a switch to fermentative metabolism. It is thought that primary or secondary plastids – organelles found in plants and algae that are responsible for producing and storing food – evolved from comparable photosynthetic endosymbionts (cyanobacteria or eukaryotic algae) that had to address these and other challenges.
